# The longitudinal relationship between night-time sleep duration, midday napping, and frailty among middle-aged and older people in China: a prospective analysis

**DOI:** 10.7189/jogh.15.04059

**Published:** 2025-02-28

**Authors:** Dongfeng Tang, Chengxu Long, Yilin Wei, Shangfeng Tang

**Affiliations:** 1 School of Life Course and Population Sciences, King’s College London, London, UK; 2 Department of Global Health and Social Medicine, King’s College London, London, UK; 3 School of Medicine and Health Management, Tongji Medical College, Huazhong University of Science and Technology, Wuhan, China; 4 Research Centre for Rural Health Service, Key Research Institute of Humanities and Social Sciences of Hubei Provincial Department of Education, Wuhan, China

## Abstract

**Background:**

Frailty is an important issue presented by ageing. Night-time sleep and midday napping are important modifiable factors influencing health, but their impacts on frailty remain unclear.

**Methods:**

We used five waves of data from the China Health and Retirement Longitudinal Study (2011–20), with 15 333 participants in the baseline sample. We used fixed effects regression models to explore longitudinal relationships between night-time sleep duration, midday napping, and frailty index (FI). We added interaction terms of sleeping and napping to the regression model to explore their combined effects. We further used the Cox proportional regression model to quantify risks for frailty.

**Results:**

Compared to sleeping seven to nine hours, sleeping <6 hours (FI = 0.016), six to seven hours (FI = 0.004), and >9 hours (FI = 0.005) were significantly associated with a mean increase in FI separately. Napping >90 minutes significantly increased FI by 0.003 compared to non-nappers. Effects of sleeping six to seven hours and >9 hours on frailty were separately enhanced by napping >90 minutes and any napping duration (except 60–90 minutes). Sleeping <6 hours and six to seven hours increased frailty risk by 44% (hazard ratio (HR) = 1.44) and 12% (HR = 1.12), respectively. Frailty risk was increased by napping >90 minutes by 14% (HR = 1.14) compared to non-nappers.

**Conclusions:**

Short (<7 hours) or long (>9 hours) sleep and prolonged midday napping (>90 minutes) were associated with frailty among the Chinese middle-aged and older population. The compensation effect of napping for short night-time sleep was not found in this study, and certain napping durations even increased risks of sleeping six to seven hours and >9 hours for frailty.

Frailty, as an important geriatric syndrome, has been considered an important public health issue worldwide, which will bring great challenges to health care systems and population health in the context of ageing [1]. It is characterised by multiple function declines and increased vulnerability to stressors and was found to be associated with many adverse health outcomes such as mortality, hospitalisation, long-term care, and falls [2,3]. Meanwhile, frailty was thought to be potentially preventable through interventions such as physical activity, home modifications, and comprehensive geriatric assessment, which makes intervention strategies to postpone the progression of frailty paramount [2,4]. Therefore, identifying modifiable risk factors could be of great significance for the management and intervention of frailty.

Great importance has been attached to the relationship between sleep-related behaviours and frailty, including sleep duration, sleep quality, midday napping, sleep-disordered breathing, daytime drowsiness, prolonged sleep latency and insomnia [5]. Long (≥9 hours) and short sleep (≤6 hours) durations were found to be associated with frailty in a cross-sectional study [6]. A meta-analysis also suggested that both long sleep duration (>8 hours, odds ratio (OR) = 1.21) and short sleep duration (<6 hours, OR = 1.13) were associated with frailty [5]. More longitudinal studies are needed to provide stronger evidence to clarify the effects of sleep duration on frailty. Midday napping is another important sleeping-related modifiable factor and very prevalent in China especially among older population [7]. The potential impacts of midday napping in terms of some adverse health outcomes, including mortality and cardiovascular diseases, have been explored [8,9]. The characteristic of broad effects of midday napping in different health outcomes make it a potential influencing factor for frailty, which is a state of cumulative decline in multiple physiologic systems [10]. However, the associations of midday napping with frailty are seldom investigated and remain unclear. Meanwhile, midday napping and night-time sleep duration can interact with each other, and midday napping is often used to compensate for poor night-time sleep although some researchers argue that excessive midday napping is harmful for nocturnal sleep [11]. The combined effects of night-time sleep duration and midday napping have already been spotted in some diseases such as type 2 diabetes and stroke [12,13]. Therefore, besides clarifying the separate association of night-time sleep and midday napping with frailty, potential combined effects also deserve attention.

In this study, we aimed to explore the longitudinal relationships between night-time sleep, midday mapping and frailty and quantified corresponding risks among the Chinese middle-aged and older population. Additionally, the combined effects of night-time sleep and midday napping were explored. Based on the aforementioned gaps, we propose the following hypotheses: both night-time sleep duration and midday napping are associated with frailty and night-time sleep duration and midday napping have combined effects on frailty.

## METHODS

### Study design and participants

In this prospective analysis, we drew data from the China Health and Retirement Longitudinal Study (CHARLS), a nationally representative investigation conducted across 450 neighbourhoods and village committees in 150 counties spanning 28 provinces. Data regarding individual demographic and socioeconomic status, health conditions, and related lifestyle information were collected among residents aged ≥45 years in China [14]. The details of the CHARLS study design and sampling have been described elsewhere [14]. Participants in this study were from a baseline survey conducted in 2011 and followed up every two or three years until 2020. A total of 17 708 individuals were successfully interviewed for the 2011 baseline CHARLS survey. Individuals were subject to exclusion based on specific criteria: those with a baseline age <45 years and individuals with missing data regarding night-time sleep duration and midday napping. We also excluded participants who had missing data from more than 20% of the variables consisting of frailty index (FI) according to a previous study [15,16]. The final sample size for fixed effects regression analysis and Cox regression analysis was 83 655 and 13 402 separately. We compared the characteristics of inclusion samples and excluded samples due to incomplete information about sleep, midday napping and FI items by wave and found the key demographics compositions (such as gender, age, hukou status, and marriage status) were similar across samples (Table S1 in the **Online Supplementary Document**).

### Measurement of frailty

We assessed frailty by FI and analysed it continuously, as well as pre-specified categories. FI was constructed based on the accumulation of health deficits, encompassing various dimensions of individuals’ well-being, such as comorbidities, cognition, mental health, and other aspects [17]. FI was computed as the ratio of present health deficits to the total potential deficits, yielding a continuous score from total fitness (zero) to total frailty (one) [16]. We adopted and adjusted a previously validated version of FI in CHARLS for this study [15] (Table S2 in the **Online Supplementary Document**). A higher FI score indicates greater frailty. We then categorised the FI score into frailty (FI≥0.25) and non-frailty (FI<0.25) for further analysis based on defined cutoff points [18].

### Measurement of night-time sleep duration

We drew self-reported night-time sleep duration from the question: ‘During the past month, how many hours of actual sleep did you get at night?’ We defined four groups (<6 hours, six to <7 hours, seven to nine hours, >9 hours per night) guided by previous studies [19]. We set as a reference group sleeping for seven to nine hours based on the recommendation from the National Sleep Foundation that suggested seven to nine hours as an ideal sleep duration for adults and aligned with previous studies [19–22].

### Measurement of midday napping

We attained self-reported midday napping from the question: ‘During the past month, how long did you take a nap after lunch?’ We categorised midday napping into five groups according to the duration of napping: non-napping, >0–30 minutes, >30–60 minutes, >60–90 minutes, and >90 minutes). 30 and 90 minutes were commonly used cut-off points to define short napping and as a suggested practice maximum for naps in previous studies [23–25].

### Covariates

We identified as covariates those variables which were found to be associated with frailty in previous studies and available in the CHARLS study. We incorporated various confounding variables, including demographic, socioeconomic, healthy lifestyle, and social engagement factors. Demographic factors contained age, gender (male *vs.* female), hukou (urban areas *vs.* rural areas), and marital status (partnered or single) [26]. Socioeconomic factors comprised education level (no formal education, elementary school or lower, middle school, and high school or above) and household capita consumption. Household capita consumption was treated as continuous and log-transformed. Healthy lifestyle factors included smoking (yes *vs.* no) and alcohol drinking (non-drinker *vs.* drinking less than once a month *vs.* drinking more than once a month) [27,28]. We defined social engagement as involvement in any type of social activities investigated in CHARLS, including interaction with friends, hobby groups, sports groups, voluntary work and so on [29].

### Statistical analysis

We presented the characteristics of participants by categories of frailty (frail *vs.* non-frail). For continuous variables, we reported means with standard deviation. For categorical variables, we reported numbers with percentages. To examine the disparity of frailty, we used the χ^2^ test or independent *t*-test. Considering the potential type I error raised by multiple tests, we performed the Bonferroni test to adjust the *P*-values.

We first considered FI as continuous and employed fixed effects models to explore the longitudinal relationships between night-time sleep duration, midday napping and FI using five waves of CHARLS data. The fixed-effects model uses each individual as their own control, effectively minimising biases caused by differences between individuals and factors that are difficult to observe. We supposed the following specification:

FI_it_ = Z_i_∂ + S_it_*β*_1_ + N_it_*β*_2_ + X_it_*β*_3_ + γ_t_ + ε_it_

FI_it_ represents the FI for individual i at year t. Z_i_ captures the fixed effects, which include all observed and unobserved time-invariant covariates. S_it_ denotes different night-time sleep duration for individuals i at year t, while N_it_ indicate different midday napping duration for individual i at year t. X_it_ accounts for time-varying control variables. γ_t_ denotes year effects, and ε_it_ is the error term. In Models 1 and 2, we estimated the crude coefficient of night-time sleep duration and midday napping separately. In Model 3, we estimated the adjusted coefficient of night-time sleep adjusted by covariates (age, gender, hukou, marriage, education, household capital consumption, alcohol drinking, smoking, and social engagement). In Model 4, we estimated the adjusted coefficient of midday napping adjusted by covariates above. In Model 5, a joint model with night-time sleep duration and midday napping included was performed after adjusting for covariates. To further explore the mutual effects of night-time sleep duration and midday napping, we created interaction terms of night-time sleep and midday napping and added them into Model 6 based on Model 5. Additionally, we also calculated the variance inflation factor and tolerance to assess multicollinearity since multiple confounders were included. No multicollinearity issue was spotted (Table S3 in the **Online Supplementary Document**). We further employed the Cox proportional hazards regression model to quantify the associations of night-time sleep duration and midday napping with frailty. Frailty, a dichotomous variable regarding the presence of frailty or not, was set as an endpoint, with the survey wave as the time scale. Those who remained as non-frail were treated as censored. The hazard ratios (HRs) with 95% confidence intervals (CIs) for frailty were reported to quantify the risks. To test the robustness of our findings, we performed the following sensitivity analyses by running fixed effects models and Cox regression models in different scenarios: 1) using alternative cut-off point 0.21 to define frailty [30]; 2) subgroups analysis by age (45–60 *vs.* ≥60 years) and gender; 3) excluding prefrail participants in baseline sample, and 4) excluding robust participants in baseline sample. We performed all analyses using Stata, version 18.0 (Stata Corp LLC, College Station, Texas, USA) and we considered a two-sided *P*-value <0.05 as significant.

### Ethics approval

The institutional review board of Peking University Health Science Centre approved the study (IRB approval number for the main household survey, including anthropometrics: IRB00001052-11015; IRB approval number for biomarker collection: IRB00001052-11014). All participants provided their written informed consent before completing the interview.

## RESULTS

### Characteristic of participants

Out of 15 333 individuals, 1931 (12.6%) were frail. A total of 7967 participants (52.0%) were female, with a mean age of 59.1 years. Of the participants, 11 833 (77.2%) were from rural areas, and 13 376 (87.2%) were in a partnered status. The largest proportion of individuals had an education level of elementary school or lower (n = 6032, 39.3%). Additionally, 11 094 (72.4%) reported no habit of drinking, while 6093 (39.7%) had a smoking habit. Social engagement was observed in 7652 participants (49.9%). Regarding night-time sleep duration, 6870 individuals (44.8%) reported sleeping between seven to nine hours per night, 4547 (29.7%) slept <6 hours and only 664 (4.3%) reported sleeping >9 hours. Midday napping habits varied, with 7170 participants (46.8%) reporting no napping, 3403 (22.2%) napping for 30–60 minutes, and 471 (3.1%) napping for 60–90 minutes. Compared with those non-frail, frail individuals were more likely to be older, female, partnered, non-smoker, live in rural residence, have an education level of elementary school or lower, drink more than once a month, have no social engagement, and sleep <6 hours (**Table 1**).

**Table 1:** Baseline characteristics of participants*

	Frailty				
**Characteristics**	**Non-frail**	**Frail**	**Total (n = 15 333)**	**t/χ2**	***P* value**
Total	13 402 (87.4)	1931 (12.6)			
Gender				80.93	<0.01
*Male*	6623 (49.4)	743 (38.5)	7366 (48.0)		
*Female*	6779 (50.6)	1188 (61.5)	7967 (52.0)		
Age in years, x̄ (SD)	58.33 (0.08)	64.43 (0.23)	59.10 (11.10)	–67.75	<0.01
Hukou				18.31	<0.01
*Urban area*	3133 (23.4)	367 (19.0)	3500 (22.8)		
*Rural area*	10 269 (76.6)	1564 (81.0)	11 833 (77.2)		
Marriage				154.76	<0.01
*Partnered*	11 862 (88.5)	1514 (78.4)	13 376 (87.2)		
*Single*	1540 (11.5)	417 (21.6)	1957 (12.8)		
Education				369.41	<0.01
*No formal education*	3385 (25.3)	831 (43.0)	4216 (27.5)		
*Elementary school or lower*	5261 (39.3)	771 (39.9)	6032 (39.3)		
*Middle school*	2915 (21.8)	219 (11.3)	3134 (20.4)		
*High school or above*	1841 (13.7)	110 (5.7)	1951 (12.7)		
Household capita consumption, x̄ (SD)	8.63 (0.01)	8.54 (0.02)	8.62 (0.87)	0.16	0.872
Alcohol drinking				130.43	<0.01
*Drink more than once a month*	2828 (21.1)	234 (12.1)	3062 (20.0)		
*Drink less than once a month*	1087 (8.1)	90 (4.7)	1177 (7.7)		
*Non-drinker*	9487 (70.8)	1607 (83.2)	11 094 (72.4)		
Smoking				16.37	<0.01
*No*	7995 (59.7)	1245 (64.5)	9240 (60.3)		
*Yes*	5407 (40.3)	686 (35.5)	6093 (39.7)		
Social engagement				107.19	<0.01
*No*	6501 (48.5)	1180 (61.1)	7681 (50.1)		
*Yes*	6901 (51.5)	751 (38.9)	7652 (49.9)		
Night sleep duration in hours				475.10	<0.01
*<6*	3585 (26.7)	962 (49.8)	4547 (29.7)		
*6–7*	2925 (21.8)	327 (16.9)	3252 (21.2)		
*7–9*	6336 (47.3)	534 (27.7)	6870 (44.8)		
*>9*	556 (4.1)	108 (5.6)	664 (4.3)		
Midday napping in minutes				2.31	0.680
*0*	6269 (46.8)	901 (46.7)	7170 (46.8)		
*0–30*	2251 (16.8)	345 (17.9)	2596 (16.9)		
*30–60*	2992 (22.3)	411 (21.3)	3403 (22.2)		
*60–90*	415 (3.1)	56 (2.9)	471 (3.1)		
*>90*	1475 (11.0)	218 (11.3)	1693 (11.0)		

SD – standard deviation, t – t value of independent *t*-test, χ2 – χ2 value of χ^2^ test, x̄ – mean

*Presented as n (%) unless specified otherwise.

### Longitudinal relationship between night-time sleep, midday napping and FI

In Model 1, compared to participants with night-time sleep duration between seven to nine hours, participants reported sleeping <6 hours (FI = 0.016; 95% CI = 0.014, 0.018, *P* < 0.001), six to seven hours (FI = 0.004; 95% CI = 0.003, 0.006, *P* < 0.001), and >9 hours (FI = 0.002; 95% CI = 0.002, 0.009, *P* = 0.001) significantly associated with a mean increase in FI. After adjusting for age, gender, hukou, marriage, education, household capita consumption, alcohol drinking, smoking, and social engagement (Model 3), the associations of sleeping <6 hours (*β* = 0.016; 95% CI = 0.014, 0.017, *P* < 0.001), six to seven hours (*β* = 0.004; 95% CI = 0.003–0.006, *P* < 0.001), and >9 hours (*β* = 0.006; 95% CI = 0.002, 0.009, *P* = 0.002) with FI remained significant, but the effect of sleeping >9 hours increased (**Table 2**).

**Table 2:** Longitudinal associations of night-time sleep, midday napping with frailty

Items	*β* (95% CI)	*P*-value
Model 1 (n = 83 979)*		
Night time sleep in hours		
*<6*	0.016 (0.014, 0.018)	<0.001
*6–7*	0.004 (0.003, 0.006)	<0.001
*>9*	0.006 (0.002, 0.009)	0.001
**Model 2 (n = 84 583)**†		
Midday day napping in minutes		
*0–30*	0.001 (–0.001, 0.003)	0.292
*30–60*	0.001 (–0.001, 0.002)	0.571
*60–90*	0.002 (–0.001, 0.004)	0.294
*>90*	0.002 (0.000, 0.005)	0.054
**Model 3 (n = 83 979)**‡		
Night time sleep in hours		
*<6*	0.016 (0.014, 0.017)	<0.001
*6–7*	0.004 (0.003, 0.006)	<0.001
*>9*	0.006 (0.002, 0.009)	0.002
**Model 4 (n = 84 583)**§		
Midday day napping in minutes		
*0–30*	0.001 (–0.001–0.003)	0.232
*30–60*	0.001 (–0.001, 0.002)	0.529
*60–90*	0.002 (–0.001, 0.005)	0.276
*>90*	0.002 (–0.000, 0.005)	0.053
**Model 5 (n = 83 655)**¶		
Night time sleep in hours		
*<6*	0.016 (0.014, 0.017)	<0.001
*6–7*	0.004 (0.003, 0.006)	<0.001
*>9*	0.005 (0.001, 0.008)	0.008
**Model 5 (n = 83 655)**		
Midday day napping in minutes		
*0–30*	0.001 (–0.001, 0.003)	0.259
*30–60*	0.001 (–0.001, 0.003)	0.271
*60–90*	0.002 (–0.001, 0.005)	0.255
*>90*	0.003 (0.000, 0.005)	0.022

CI – confidence interval

*Includes night-time sleep duration.

†Includes midday napping.

‡Includes night-time sleep duration after adjusting for age, gender, hukou, marriage, education, household capital consumption, alcohol drinking, smoking, and social engagement.

§Includes midday napping after adjusting for age, gender, hukou, marriage, education, household capital consumption, alcohol drinking, smoking, and social engagement.

¶Includes both night-time sleep duration and midday napping after adjusting for age, gender, hukou, marriage, education, household capital consumption, alcohol drinking, smoking, and social engagement.

In Model 2, the associations of any group of midday napping with FI were consistently not significant compared to non-nappers before and after controlling covariates (Model 4). Napping >90 minutes was associated with a mean increase in FI of 0.002 (Model 2 95% CI = 0.001, 0.005; Model 4 95% CI = –0.001, 0.005) both in Model 2 and Model 4, although not significant (*P* = 0.054 and *P* = 0.053). In the joint model (Model 5), we included both night-time sleep duration and midday napping after adjusting for all covariates. Compared to sleeping between seven to nine hours, sleeping <6 hours, six to seven hours, and >9 hours was significantly associated with a mean increase in FI of 0.016 (95% CI = 0.014, 0.017, *P* < 0.001), 0.004 (95% CI = 0.003–0.006, *P* < 0.001), and 0.005 (95% CI = 0.001, 0.008, *P* = 0.008) separately. Compared to non-nappers, napping >90 minutes also significantly increased FI by 0.003 (95% CI = 0.001, 0.005, *P* = 0.022).

There were significant interactions among subgroups of sleeping six to seven hours with napping >90 minutes (*β* = 0.005; *P* = 0.026), sleeping >9 hours with napping zero to 30 minutes (*β* = 0.016; *P* = 0.004), sleeping >9 hours with napping 30–60 minutes (*β* = 0.010; *P* = 0.028), and sleeping >9 hours with napping >90 minutes (*β* = 0.012; *P* < 0.001) (**Table 3**).

**Table 3:** The interaction effects of night-time sleep and midday napping*

	Total (n = 83 655)		
**Items**	***β***	**95% CI**	***P*-value**
Night time sleep <6 hours and midday napping in minutes			
*0–30*	<0.001	(–0.004, 0.004)	0.968
*30–60*	0.001	(–0.002, 0.005)	0.562
*60–90*	–0.003	(–0.009, 0.004)	0.390
*>90min*	–0.001	(–0.006, 0.004)	0.700
Night time sleep 6–7 hours and midday napping in minutes			
*0–30*	0.001	(–0.002, 0.005)	0.440
*30–60*	0.001	(–0.002, 0.005)	0.510
*60–90*	–0.002	(–0.008, 0.004)	0.598
*>90min*	0.005	(0.001, 0.010)	0.026
Night time sleep >9 hours and midday napping in minutes			
*0–30*	0.016	(0.005, 0.027)	0.004
*30–60*	0.010	(0.001, 0.018)	0.028
*60–90*	0.011	(–0.003, 0.026)	0.131
*>90min*	0.021	(0.012, 0.030)	<0.001

CI – confidence interval

*The model, including interaction terms, was adjusted for night-time sleep duration, midday napping, age, gender, hukou, marriage, education, household capital consumption, alcohol drinking, smoking, and social engagement.

### The risks of night-time sleep duration and midday napping

**Figure 1:**
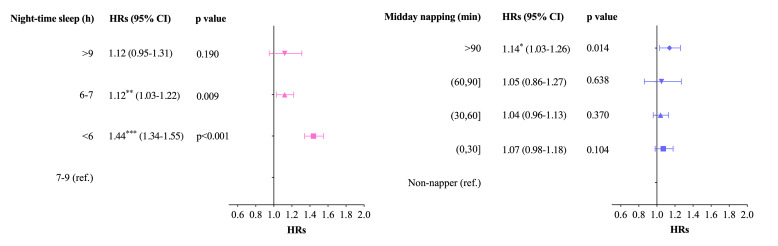
Hazards of night-time sleep duration and midday napping. CI – confidence interval, HR – hazard ratio, ref – reference.

In the Cox regression model with night-time sleep duration and midday napping after controlling covariates (age, gender, hukou, marriage, education, household capital consumption, alcohol drinking, smoking, and social engagement), night-time sleep duration and midday napping were identified to be associated with frailty risks (**Figure 1**). Compared with sleeping seven to nine hours, there were relative risks of developing frailty with sleeping six to seven hours (HR = 1.12; 95% CI = 1.03, 1.22) and sleeping <6 hours (HR = 1.44; 95% CI = 1.34, 1.55). Compared with non-nappers, napping >90 minutes increased the risk for frailty by 14% (HR = 1.14; 95% CI = 1.03, 1.26).

### Sensitivity analyses

The main findings of our analyses remain consistent in sensitivity analyses (Table S4–10 in the **Online Supplementary Document**). Of note, although the associations were not significant, napping 30–60 minutes, napping 60–90 minutes, and napping >90 minutes were found to be associated with a mean decrease in FI = 0.001 among participants aged 45–60 years (Table S5 in the **Online Supplementary Document**). The repeated Cox regression analysis among a subgroup of individuals aged 45–60 years also had similar results and found napping 30–60 minutes (HR = 0.95; *P* = 0.982) and 60–90 minutes (HR = 0.99; *P* = 0.643) non-significantly associated with lower risk of frailty (Table S6 in the **Online Supplementary Document**).

## DISCUSSION

We found that unnormal night-time sleep duration (<6 hours, six to seven hours, and >9 hours) and napping >90 minutes were significantly associated with higher FI. Unnormal night-time sleep duration played as independent risk factors across study waves and the effect could be moderated by midday napping. Compared with individuals sleeping seven to nine hours, there were higher risks for those sleeping <6 hours (HR = 1.44) and six to seven hours (HR = 1.12) to develop frailty. Napping >90 minutes (HR = 1.14) also had a higher risk for onset of frailty against non-nappers.

Sleeping <6 hours, six to seven hours, and >9 hours were identified as independent risk factors for higher frailty index which was a biomarker for accumulation of health deficits. Additionally, our study further suggested that sleeping <6 hours and six to seven hours separately increased 44% and 12% risk for frailty compared with sleeping seven to nine hours. Previous studies also supported our findings [6,31]. Compared to the existing literature, we successfully provided stronger evidence because of the advantages of sample size and cohort study design. We also found napping >90 minutes was longitudinally associated with higher FI and could increase 14% of risk for frailty compared to non-nappers. Only one relevant study existed to our best knowledge and found napping <30 minutes increased the risk for frailty after investigating 5126 participants [32]. But it adopted Physical Frailty Phenotype (PFP) as the measurement of frailty, which is different from FI we used. It is not appropriate to treat PFP and FI alternative since they have different application scope. PFP defines frailty as a pre-disability syndrome and therefore meaningful results are potentially restrict to non-disable older population. But FI apply to individuals regardless of function status or age [33]. Furthermore, FI as an continues score, is more sensitive for modifications than categorial PFP, which makes associated factors easier to be told [33]. Additionally, a longer follow-up period and larger sample size in our study also added value in exploring risk of midday napping for frailty on the basis of the precious study.

However, the spotted association of midday napping with the frailty index needs a cautious interpretation since the coefficient was significant only when night-time sleep duration was added into the model, which suggested the effect of midday napping potentially interacted with night-time sleep. This hypothesis was confirmed by our following interaction terms analysis. Additionally, our sensitivity analysis also found that midday napping 30–60 minutes and 60–90 minutes could potentially play as protective factors among the middle-aged population, although the associations were not significant. More studies are needed to clarify the effects of midday napping among populations of different age groups.

The mechanisms underlying associations of sleep duration and midday napping with frailty remain to be explored. In this study, frailty was measured by the accumulation of health deficits, including comorbidities, self-rating health, basic activities of daily living (ADLs) and instrumental ADLs (IADLs), mental health, and cognition. The mechanisms could be partly explained by the significant associations with components of FI. Previous studies have identified short or prolonged night-time sleep duration and midday napping as risk factors for comorbidity conditions such as hypertension, type 2 diabetes, heart disease, stroke, and asthma [34–36]. Regarding self-rating health, a systematic review concluded that extreme sleep duration (short or long) was related with worse self-rated health [37]. Arthur Eumann Mesas found long sleep duration was a marker of functional limitations measured by IADL [38]. Furthermore, both sleep duration and midday napping were found to associated with depression [39,40]. Additionally, both extreme night-time sleep duration and midday napping duration were linked to poorer cognition [23,41]. Therefore, the impacts of night-time sleep duration and midday napping on various health outcomes might lead to the development of frailty. In previous studies predominated by frailty phenotype, some hypothesizes could help to understand the mechanisms. Disruptions in neuroendocrine regulation brought by short sleep duration including reductions in testosterone levels, chronic inflammation, and greater oxidative stress levels could contribute to frailty risk [31,42]. The risk of long sleep duration could potentially be explained by elevated melatonin and cortisol levels, a lower body temperature and less physical activities which were linked to frailty [43–45]. Additionally, the risk of midday napping could also partly be explained by cultural factors behind midday napping behaviour. Midday napping was often regarded as a compensation for poor night-time sleep, and this point is especially popular among the older population in the Chinese context [11]. Therefore, those having a habit of midday napping are more likely to have poor night-time sleep and to be older people, which are risk factors for frailty.

We found napping >90 minutes would increase the risk of sleeping six to seven hours for frailty, and any duration of midday napping except napping 60–90 minutes might amplify the risk of sleeping >9 hours. One of the motivations for habitual nappers was using midday napping to compensate for short night-time sleep [11]. However, taking a prolonged midday napping (>90 minutes) among individuals with six to seven hours sleep duration are found to be risky based on our results. One of the mechanisms behind might be decreased same-day night-time sleep duration and sleep quality brought by napping. Previous studies found prolonged napping would lead to shorter same-day night-time sleep duration and poorer sleep quality (lower sleep efficiency and increased sleep fragmentation), which would further increase the risk for frailty of short sleepers [46,47]. Additionally, both short sleep and napping were spotted having a link with inflammation, which brought an elevated frailty risk [31,46]. Meanwhile, napping after sleeping >9 hours are not advisable according to our study, either. Therefore, we should take a more cautious attitude towards midday napping and avoid prolonged night-time sleep duration and midday napping.

To prevent the onset of frailty and postpone its development, maintaining a healthy sleeping pattern is essential, which is to have recommended night-time sleep duration (seven to nine hours) and avoid napping >90 minutes. For individuals with short night-time sleep duration, the potential harm cannot be eliminated by adding midday napping duration based on our results. On the contrary, over-long napping duration would elevate the risk for frailty. Additionally, any duration of midday napping is not advisable for individuals sleeping >9 hours.

In this study, we first analysed the longitudinal associations of night-time sleep duration and midday napping with frailty index simultaneously and quantified their risks for onset of frailty, using a nationally representative sample across nine years. But some limitations existed. First, night-time sleep duration and midday napping relied on self-reported information, which introduced recall bias. For example, many older people might often nap but without realizing it, which would potentially lead to an underestimation of napping risk. Future studies using objective measures are needed to validate the results of this study. Second, although fixed effects models were used to eliminate the heterogeneity brought by time-varying confounders and some of them had been controlled in our models, some other time-varying covariates might exist. Additionally, due to the data availability, some key variables were omitted in this study such as some sleep-related factors (sleep quality, and sleep disorders) and physical activity. Future studies using robust models with a more comprehensive covariates span are recommended. Third, both night-time sleep duration and midday napping exhibit dynamic patterns and may follow distinct trajectories. Our study failed to clarify the effects of trajectories of night-time sleep and midday napping. Additionally, although we took the combined effects of night-time sleep duration and midday napping into consideration, the real sleep patterns might be more complex, requiring considering more factors such as sleep quality. Studies are needed to understand the impacts of sleep and napping trajectories and define more complete healthy sleep patterns. Fourthly, selection bias might be introduced when excluding participants without complete information regarding sleep, midday napping and FI items. For example, excluded participants are more likely to have social engagement, which is a protective factor for frailty in our study. This selection might make our results underestimate the risk of night-time sleep duration and midday napping, which requires a more cautious interpretation of results. At last, the underlying mechanisms for the pathways between night-time sleep, midday napping and frailty are still vague, requiring more experimental studies.

## CONCLUSIONS

In conclusion, this study found sleeping <7 hours or >9 hours and napping >90 minutes were associated with frailty among Chinese middle-aged and older population. Additionally, our results failed to provide evidence for the point that midday napping could compensate for the health loss brought about by a short sleep. On the contrary, we found midday napping even elevated the risk for frailty among individuals with six to seven hours or >9 hours of night-time sleep duration. It is recommended to maintain a healthy sleep pattern by ensuring a night-time sleep duration of seven to nine hours and avoiding prolonged midday napping exceeding 90 minutes.

## Additional material


Online Supplementary Document
