# Supplementary material for: The longitudinal relationship between night-time sleep duration, midday napping, and frailty among middle-aged and older people in China: a prospective analysis
Source: J Glob Health. 2025 Feb 28;15:04059. doi: 10.7189/jogh.15.04059 (PMC11869517; doi:10.7189/jogh.15.04059)
Supplement: Online Supplementary Document [file jogh-15-04059-s001.pdf]

Supplement to: Tang D, Long C, Wei Y, Tang S. The longitudinal relationship between night-time sleep duration, midday napping, and frailty among middle-aged and older people in China: a prospective analysis. J Glob Health. 2025;15:04059.

Online Supplementary document

Table S1. Characteristics of exclusion samples and inclusion samples

		2011		2013		2015		2018		2020	
		Inclusion (N=15333)	Exclusion (N=1773)	Inclusion (N=15217)	Exclusion (N=2879)	Inclusion (N=15354)	Exclusion (N=7088)	Inclusion (N=19204)	Exclusion (N=316)	Inclusion (N=18547)	Exclusion (N=586)
Gender (N, %)											
	Male	7366 (48%)	979 (55.2%)	7384 (48.5%)	1402 (48.7%)	7416 (48.3%)	2857 (40.3%)	9167 (47.7%)	138 (43.7%)	8836 (47.6%)	214 (36.5%)
	Female	7967 (52%)	794 (44.8%)	7833 (51.5%)	1477 (51.3%)	7938 (51.7%)	4231 (59.7%)	10037 (52.3%)	178 (56.3%)	9711 (52.4%)	372 (63.5%)
Age (Mean，SD)		59.10 (9.70)	59.20 (11.10)	59.71 (9.43)	61.08 (12.01)	60.99 (9.25)	58.24 (10.21)	61.90 (10.07)	51.93 (9.83)	61.66 (9.74)	66.86 (11.62)
Hukou (N, %)											
	Urban area	3500 (22.8%)	362 (20.4%)	3484 (22.9%)	778 (27.0%)	3138 (20.4%)	1286 (18.1%)	4045 (21.1%)	73 (23.1%)	4770 (25.7%)	101 (17.2%)
	Rural area	11833 (77.2%)	1411 (79.6%)	11733 (77.1%)	2101 (73.0%)	12216 (79.6%)	5802 (81.9%)	15159 (78.9%)	243 (76.9%)	13777 (74.3%)	485 (82.8%)
Marriage (N, %)											
	Partnered	13376 (87.2%)	1487 (83.9%)	13337 (87.7%)	2348 (81.6%)	13294 (86.6%)	6301 (88.9%)	16308 (84.9%)	283 (89.6%)	15622 (84.2%)	391 (66.7%)
	Single	1957 (12.8%)	286 (16.13%)	1880 (12.4%)	531 (18.4%)	2060 (13.4%)	787 (11.1%)	2896 (15.1%)	33 (10.4%)	2925 (15.8%)	195 (33.3%)
Education (N, %)											
	No formal education	4216 (27.5%)	517 (29.2%)	3840 (25.2%)	951 (33.0%)	3834 (25.0%)	1233 (17.4%)	4694 (24.4%)	49 (15.5%)	4004 (21.6%)	244 (41.6%)
	Elementary school or lower	6032 (39.3%)	637 (35.9%)	6202 (40.8%)	929 (32.3%)	6290 (41.0%)	4544 (64.1%)	8630 (44.9%)	214 (67.7%)	7963 (42.9%)	231 (39.4%)
	Middle school	3134 (20.4%)	387 (21.8%)	3239 (21.3%)	568 (19.7%)	3336 (21.7%)	750 (10.6%)	3680 (19.2%)	30 (9.5%)	4147 (22.4%)	76 (13.0%)
	High school or above	1951 (12.7%)	232 (13.1%)	1936 (12.7%)	431 (15.0%)	1894 (12.3%)	561 (7.9%)	2200 (11.5%)	23 (7.3%)	2433 (13.1%)	35 (6.0%)

Household capita consumption (Mean，SD)		8.62 (0.87)	8.42 (0.89)	8.91 (0.87)	8.99 (0.89)	9.08 (0.93)	9.29 (0.94)	9.36 (0.95)	9.66 (0.91)	9.50 (0.96)	9.37 (1.09)
Drink (N, %)											
	Non-drinker	3062 (20%)	45 (2.5%)	4086 (26.9%)	633 (22.0%)	4030 (26.3%)	1493 (21.1%)	12727 (66.3%)	227 (71.8%)	4969 (26.8%)	92 (15.7%)
	Drink less than once a month	1177 (7.7%)	158 (8.9%)	1197 (7.9%)	234 (8.1%)	1307 (8.5%)	480 (6.8%)	1446 (7.5%)	17 (5.4%)	1759 (9.5%)	40 (6.8%)
	Drink more than once a month	11094 (72.4%)	1570 (88.6%)	9934 (65.3%)	2012 (69.9%)	10017 (65.2%)	5115 (72.2%)	5031 (26.2%)	72 (22.8%)	11819 (63.7%)	454 (77.5%)
smoke (N, %)											
	No	9240 (60.3%)	1029 (58.0%)	8686 (57.1%)	1689 (58.7%)	8485 (55.3%)	4689 (66.2%)	11019 (57.4%)	235 (74.4%)	11270 (60.8%)	415 (70.8%)
	Yes	6093 (39.7%)	744 (42.0%)	6531 (42.9%)	1190 (41.3%)	6869 (44.7%)	2399 (33.8%)	8185 (42.6%)	81 (25.6%)	7277 (39.2%)	171 (29.2%)
Social engagement (N, %)											
	No	7681 (50.1%)	361 (20.4%)	6331 (41.6%)	550 (19.1%)	7098 (46.2%)	1967 (27.8%)	9063 (47.2%)	79 (25.0%)	9546 (51.5%)	417 (71.2%)
	Yes	7652 (49.9%)	1412 (79.6%)	8886 (58.4%)	2329 (80.9%)	8256 (53.8%)	5121 (72.2%)	10141 (52.8%)	237 (75.0%)	9001 (48.5%)	169 (28.8%)

Table S2. Frailty index items

No.	Items
1	Presence of hypertension.
2	Presence of dyslipidemia.
3	Presence of diabetes or high blood glucose.
4	Presence of chronic lung diseases.
5	Presence of liver disease
6	Presence of heart disease
7	Presence of stroke
8	Presence of kidney disease
9	Presence of stomach or other digestive diseases.
10	Presence of emotional, nervous, or psychiatric problems.
11	Presence of memory-related disease
12	Presence of arthritis or rheumatism.
13	Presence of asthma
14	Self-rating of health
15	Limitations in dressing
16	Limitations in eating
17	Limitations in bathing or showering
18	Limitations in getting into or out of bed.
19	Limitations in using the toilet
20	Difficulty with controlling urination and defecation.
21	Limitations in doing household chores.
22	Limitations in preparing hot meals.
23	Limitations in shopping.
24	Limitations in managing money
25	Limitations in taking medications

36	Presence of depressive symptom (the score of CESD-10>10)
27	Cognitive decline (1-(the cognition score/21))

Measurement of self-rating health: Self-rated health was assessed by asking participants to rate their health status on a scale of 1-5 (very good, good, fair, poor, or very poor). Then the scores were inverse recoded (very good=0, good=0.25, fail=0.5, poor=0.75, very poor=1). CESD-10: The 10-item Center for Epidemiologic Studies Depression Scale. Measurement of cognition score: It was measured by Telephone Interview of Cognitive Status-10 (TICS-10), word recall, and figure drawing. And the summary score of cognition ranges from 0 to 21.

Table S3 Variance inflation factor of covariates

	VIF	Tolerance
Gender	2.25	0.44
Age	1.29	0.78
Hukou	1.25	0.80
Marriage	1.14	0.88
Education	1.45	0.69
Household capita consumption	1.21	0.82
Drink	1.16	0.87
smoke	2.05	0.49
Social engagement	1.05	0.95
Night-sleep duration	1.02	0.98
Midday napping	1.04	0.96

VIF<10, and Tolerance>0.1 were regarded as having no multicollinearity issue.

Table S4. Sensitivity analysis by using 0.21 as cut-off point of FI: hazards of night-time sleep duration and midday napping

	HR	95% CI		P value
		lower	upper	
Night time sleep (ref.7-9h)				
<6h	1.37	1.28	1.46	p<0.001
[6,7)	1.10	1.01	1.18	0.022
>9h	1.08	0.93	1.26	0.315
Midday day napping (ref. Non naaping)				
(0, 30]min	1.08	1.00	1.18	0.056
(30,60]min	1.04	0.96	1.12	0.307
(60,90] min	1.02	0.86	1.22	0.802
>90min	1.13	1.03	1.25	0.012

The model was adjusted for age, gender, hukou, marriage, education, household capital consumption, alcohol drinking, smoking, and social engagement.

Table S5. Sensitivity analysis by age: longitudinal associations of night-time sleep, midday napping with frailty

	Age:45-60				Age: ≥60			
	β coefficient	95% CI		P value	β coefficient	95% CI		P value
		lower	upper			lower	upper	
Night time sleep (ref.7-9h)								
<6h	0.013	0.011	0.015	p<0.001	0.017	0.014	0.020	p<0.001
[6,7)	0.004	0.003	0.006	p<0.001	0.005	0.002	0.007	p<0.001
>9h	0.002	-0.003	0.006	0.475	0.005	0.000	0.010	0.076
Midday day napping (ref. Non naaping)								
(0, 30]min	0.001	-0.001	0.004	0.239	0.002	-0.001	0.005	0.210
(30,60]min	-0.001	-0.002	0.002	0.952	0.002	-0.001	0.005	0.170
(60,90] min	-0.001	-0.004	0.003	0.694	0.003	-0.002	0.007	0.282
>90min	-0.001	-0.003	0.002	0.677	0.004	0.001	0.008	0.022

The model was adjusted for age, gender, hukou, marriage, education, household capital consumption, alcohol drinking, smoking, and social engagement.

Table S6. Sensitivity analysis by age: hazards of night-time sleep duration and midday napping

	Age: 45-60				Age: ≥60			
	HR	95% CI		P value	HR	95% CI		P value
		lower	upper			lower	upper	
Night time sleep (ref.7-9h)								
<6h	1.50	1.35	1.68	p<0.001	1.37	1.25	1.51	p<0.001
[6,7)	1.15	1.01	1.30	0.031	1.08	0.96	1.22	0.194
>9h	1.12	0.88	1.43	0.357	1.14	0.92	1.41	0.244
Midday day napping (ref. Non naaping)								
(0, 30]min	1.03	0.90	1.18	0.513	1.11	0.98	1.25	0.096
(30,60]min	0.95	0.84	1.08	0.982	1.12	1.01	1.25	0.040
(60,90] min	0.99	0.74	1.33	0.643	1.09	0.84	1.41	0.510
>90min	1.08	0.91	1.27	0.291	1.19	1.04	1.37	0.010

The model was adjusted for age, gender, hukou, marriage, education, household capital consumption, alcohol drinking, smoking, and social engagement.

Table S7. Sensitivity analysis by gender: longitudinal associations of night-time sleep, midday napping with frailty

	Gender: male				Gender: female			
	β coefficient	95% CI		P value	β coefficient	95% CI		P value
		lower	upper			lower	upper	
Night time sleep (ref.7-9h)								
<6h	0.015	0.013	0.018	p<0.001	0.016	0.013	0.018	p<0.001
[6,7)	0.004	0.002	0.006	0.001	0.005	0.003	0.007	p<0.001
>9h	0.006	0.001	0.011	0.023	0.004	-0.001	0.009	0.141
Midday day napping (ref. Non naaping)								
(0, 30]min	0.001	-0.002	0.003	0.705	0.001	-0.001	0.004	0.295
(30,60]min	-0.001	-0.003	0.002	0.610	0.002	0.000	0.005	0.063
(60,90] min	0.000	-0.004	0.004	0.927	0.003	-0.001	0.008	0.181
>90min	0.002	-0.001	0.006	0.17	0.003	-0.001	0.006	0.105

The model was adjusted for age, gender, hukou, marriage, education, household capital consumption, alcohol drinking, smoking, and social engagement.

Table S8. Sensitivity analysis by gender: hazards of night-time sleep duration and midday napping

	Sex: male				Sex: female			
	HR	95% CI		P value	HR	95% CI		P value
		lower	upper			lower	upper	
Night time sleep (ref.7-9h)								
<6h	1.32	1.17	1.49	p<0.001	1.53	1.39	1.68	p<0.001
[6,7)	1.07	0.94	1.22	0.297	1.16	1.04	1.30	0.010
>9h	1.13	0.89	1.44	0.325	1.09	0.88	1.36	0.426
Midday day napping (ref. Non naaping)								
(0, 30]min	1.14	0.98	1.32	0.081	1.04	0.93	1.17	0.484
(30,60]min	1.03	0.91	1.17	0.63	1.06	0.95	1.18	0.328
(60,90] min	1.18	0.92	1.53	0.191	0.89	0.66	1.21	0.456
>90min	1.16	0.99	1.35	0.062	1.13	0.98	1.31	0.095

The model was adjusted for age, gender, hukou, marriage, education, household capital consumption, alcohol drinking, smoking, and social engagement.

Table S9. Sensitivity analysis by baseline status: longitudinal associations of night-time sleep, midday napping with frailty

	Baseline status: robust				Baseline status: prefrail			
	β coefficient	95% CI		P value	β coefficient	95% CI		P value
		lower	upper			lower	upper	
Night time sleep (ref.7-9h)								
<6h	0.010	0.008	0.013	p<0.001	0.019	0.016	0.022	p<0.001
[6,7)	0.002	0.001	0.004	0.007	0.006	0.004	0.008	p<0.001
>9h	0.001	-0.003	0.006	0.630	0.008	0.003	0.013	0.003
Midday day napping (ref. Non naaping)								
(0, 30]min	0.003	0.000	0.005	0.025	0.000	-0.003	0.003	0.999
(30,60]min	0.003	0.001	0.005	0.010	0.000	-0.003	0.002	0.760
(60,90] min	0.002	-0.001	0.006	0.207	0.001	-0.003	0.006	0.612
>90min	0.004	0.001	0.007	0.007	0.002	-0.002	0.005	0.298

The model was adjusted for age, gender, hukou, marriage, education, household capital consumption, alcohol drinking, smoking, and social engagement.

Table S10. Sensitivity analysis by baseline status: hazards of night-time sleep duration and midday napping

	Baseline status: robust				Baseline status: prefrail			
	HR	95% CI		P value	HR	95% CI		P value
		lower	upper			lower	upper	
Night time sleep (ref.7-9h)								
<6h	1.13	0.94	1.37	0.19	1.23	1.14	1.34	p<0.001
[6,7)	1.10	0.91	1.33	0.327	1.06	0.96	1.17	0.243
>9h	1.37	0.98	1.90	0.065	0.97	0.81	1.17	0.785
Midday day napping (ref. Non naaping)								
(0, 30]min	1.07	0.86	1.31	0.553	1.06	0.96	1.17	0.258
(30,60]min	0.90	0.73	1.10	0.286	1.02	0.94	1.12	0.629
(60,90] min	1.19	0.79	1.79	0.405	0.99	0.80	1.23	0.931
>90min	1.10	0.87	1.40	0.432	1.13	1.00	1.26	0.042

The model was adjusted for age, gender, hukou, marriage, education, household capital consumption, alcohol drinking, smoking, and social engagement.
